# Multi-Modal Segmentation of 3D Brain Scans Using Neural Networks

**DOI:** 10.3389/fneur.2021.653375

**Published:** 2021-07-14

**Authors:** Jonathan Zopes, Moritz Platscher, Silvio Paganucci, Christian Federau

**Affiliations:** Institute for Biomedical Engineering, ETH Zürich, Zurich, Switzerland

**Keywords:** brain imaging (CT and MRI), anatomical segmentation, multi-modal, convolutional neural networks, dropout sampling

## Abstract

Anatomical segmentation of brain scans is highly relevant for diagnostics and neuroradiology research. Conventionally, segmentation is performed on *T*_1_-weighted MRI scans, due to the strong soft-tissue contrast. In this work, we report on a comparative study of automated, learning-based brain segmentation on various other contrasts of MRI and also computed tomography (CT) scans and investigate the anatomical soft-tissue information contained in these imaging modalities. A large database of in total 853 MRI/CT brain scans enables us to train convolutional neural networks (CNNs) for segmentation. We benchmark the CNN performance on four different imaging modalities and 27 anatomical substructures. For each modality we train a separate CNN based on a common architecture. We find average Dice scores of 86.7 ± 4.1% (*T*_1_-weighted MRI), 81.9 ± 6.7% (fluid-attenuated inversion recovery MRI), 80.8 ± 6.6% (diffusion-weighted MRI) and 80.7 ± 8.2% (CT), respectively. The performance is assessed relative to labels obtained using the widely-adopted FreeSurfer software package. The segmentation pipeline uses dropout sampling to identify corrupted input scans or low-quality segmentations. Full segmentation of 3D volumes with more than 2 million voxels requires <1s of processing time on a graphical processing unit.

## 1. Introduction

Anatomical segmentation of magnetic resonance imaging (MRI) or computed tomography (CT) scans is important for clinical diagnostics and scientific research. In particular, quantitative volumetric measures of anatomical structures can be derived from accurate segmentation labels, which can then be used to identify and monitor the progression of degenerative diseases, such as Alzheimer's disease, which is characterized by atrophy of the hippocampus and the medial temporal lobe (1), Huntington disease, which results in athrophy of the striatum (2), and frontotemporal lobar degeneration, which causes atrophy of the frontal and temporal lobes (3).

Manual brain segmentation, however, requires expert knowledge of radiologists, is extremely tedious and time consuming, and is therefore limited to small datasets or simply not available. An alternative approach is to automatize segmentation, which sparked the development of various segmentation software packages. In brain imaging these include e.g., FreeSurfer (4), BrainSuite (5), FSL (6), and ANTS (7). These tools apply a set of complex transformations and thresholding procedures to the input volume (8) and are typically tailored toward *T*_1_-weighted scans. As a consequence, direct segmentation of highly relevant MRI contrasts like FLAIR (fluid-attenuated inversion recovery) or DWI (diffusion-weighted imaging) remain unsupported. The same statement is true for CT volumes.

Although the recent literature contains attempts to automatize segmentation on FLAIR (9–11), DWI (12, 13), or CT volumes (14, 15), a comparative study on the achievable segmentation quality on the different imaging modalities is, to the best of our knowledge, still outstanding. We attribute this in part to the lack of structured databases that contain several paired imaging modalities for the same patient. Further, the limited flexibility of conventional segmentation tools, that require careful fine-tuning of parameters, might be a second contributing factor.

In our work, we present a broad study on the segmentation performance achievable on *T*_1_-weighted MRI, FLAIR, DWI, and CT scans for a wide range of 27 anatomical classes. The analysis is based on two large databases with in total 853 MRI/CT scans and with several imaging modalities per patient. To implement a flexible segmentation pipeline, which can be quickly adapted to the different imaging modalities, we leverage the flexibility and performance of convolutional neural networks (CNNs).

**Figure 1:**
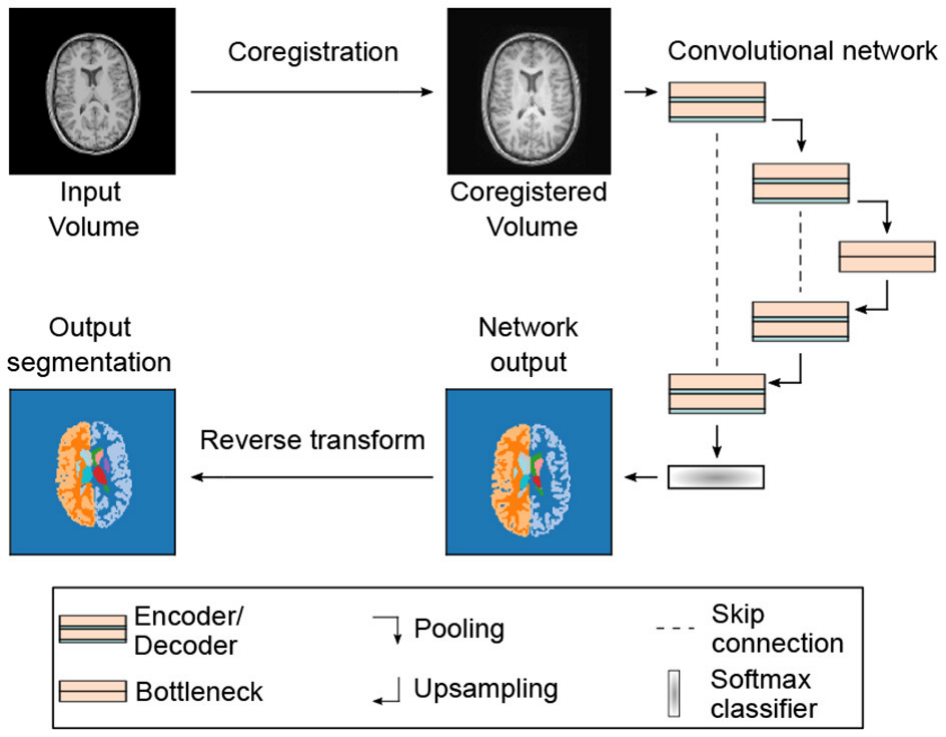
Segmentation pipeline and neural network architecture: 3D MRI or CT input volumes are coregistered to a reference volume with an affine transformation. By proper resampling the pixel dimensions of the registered volume are adjusted to the input shape of the neural network. In addition, the pixel intensities are normalized to the interval *I* = [0, 100]. Neural networks are based on the U-Net architecture (22) with 3D convolutions in the encoder and decoder blocks. Each encoder and decoder block contains two consecutive convolution, batch normalization and rectified linear activation operations. The encoder and decoder blocks are followed by a dropout layer (see Table 1 and text for details). The softmax output of the network is converted into a segmentation map with 28 labels (including background). The segmentation map is finally registered back to the input volume using the inverse affine transformation of the initial coregistration.

The recent success of CNNs in computer vision tasks (16) provided a strong impetus for applying CNNs in brain segmentation (17–21). CNNs can be rapidly adjusted to segment on a given contrast, merely by adjusting the weights of the neural network via training. This eliminates the need for additional human fine-tuning and enables us to benchmark the segmentation performance for a common network architecture (see Figure 1). Further, CNN segmentation tools recently exceeded conventional processing tools in performance (23, 24) and due to their efficient implementation on graphical processing units (GPUs), achieve full segmentation of 3D volumes almost in real-time. This is orders of magnitude faster than with conventional methods (25).

## 2. Methods

### 2.1. Segmentation Pipeline

In Figure 1, we show a schematic of our segmentation pipeline. The input MRI/CT volume is first coregistered to a reference volume with an affine transformation. The reference volume was selected from our data set by optimizing signal-to-noise ratio and by ensuring the absence of imaging artifacts. For coregistration we use the registration tool elastix 4.8 (26). The coregistered volume is resampled using spline interpolation to match the input dimensions of the segmentation CNN. The coregistration procedure increases the performance of the segmentation network and further allows for arbitrarily shaped input volumes due to resampling.

For segmentation we use a fully-convolutional neural network (F-CNN) based on the U-Net architecture (22). A schematic of the network architecture is displayed in Figure 1 and further details on network training and parameters are discussed in the subsequent sections. The network outputs a softmax quasi-probability map *P*_*s*_(*x*) for each segmentation class *s* ∈ S. Each individual map has the same dimension as the input image. The list of segmented classes S follows reference (23) and comprises in total 27 structures. All segmented classes are listed in Supplementary Table 1.

The softmax output *P* of the network is converted to a hard segmentation mask *S* using the *arg* max function:

(1) $$\begin{array}{r} S\left(x\right)=\text{arg }\underset{s}{\max}P_{s}\left(x\right). \end{array}$$

Subsequently, the hard segmentation mask *S* is registered back to the input volume. For this purpose the initial affine coregistration transformation is inverted. After applying the inverse transformation the mask is resampled using nearest-neighbor sampling with the dimensions defined by the initial input volume.

### 2.2. Neural Networks and Training

As mentioned before, we use a U-Net based network architecture for segmentation. Following the findings in (27), we make only minor modifications to the original implementation in (22, 28). The network consists of an encoder-decoder structure with skip connections (see Figure 1). In each encoder and decoder block we apply two repetitions of convolutional layers, with kernel size *K* = (3, 3, 3). Each convolutional layer is followed by batch normalization and non-linear activation with rectified linear units. The initial number of feature maps, after the first convolutional layer, was fixed to *F* = 32 for all models and after each encoder (decoder) block the number of feature maps is doubled (halved).

We use dropout layers after the encoders and decoders to prevent overfitting and to perform dropout sampling for uncertainty quantification (see section 3.3). Max pooling after each encoder block halves the feature map dimensions. Likewise, upsampling with transpose convolutions after the decoder blocks doubles the feature map dimensions and finally restores the initial dimensions at the output.

The number of max pooling operations defines the depth *D* of the U-Net architecture, which we fixed to *D* = 4 for all trained models. The bottleneck block restricts information flow from encoder to decoder and consists of two convolutional layers, each followed by batch normalization and rectified linear activation. In contrast to the encoder and decoder blocks, we do not use dropout layers in the bottleneck block (29).

CNNs are implemented in tensorflow 2.2.0 (30) and training is performed on a single GPU (Nvidia Titan RTX 24GB). Due to memory constraints the input brain volumes are limited to about 2 million voxels, which we typically distribute evenly among the imaging dimensions. The input dimensions for each network are listed in Table 1. We train the network using the Adam optimizer with initial learning rates of 0.001. During training, we apply a set of random transformations, e.g., translations, rotations, or cropping, to the volumes for data augmentation. As the loss function, we use a combination of the Dice score, summed over all class labels, and the categorical cross-entropy function:

(2) $$ℒ=-\sum_{s\in S}\left(\frac{2\sum_{x}P_{s}(x)T_{s}(x)}{\sum_{x}P_{s}(x)+T_{s}(x)}-\sum_{x}T_{s}(x)\log(P_{s}(x))\right).$$

**Table 1:** Parameters of training and test datasets and segmentation scores on all four imaging modalities using all available training samples.

**Modality**	***N*_train_**	***N*_val_**	***N*_test_**	**Volume shape**	**Average** $D_{A}$	**Weighted** $D_{V}$	$C\left(D_{A},CV\right)$	**ASSD [mm]**
MPRAGE	465	51	6	[128, 128, 128]	(86.7 ± 4.1)%	(88.7 ± 0.8)%	−0.91	(0.67 ± 0.23)
FLAIR	107	11	6	[128, 128, 128]	(81.9 ± 6.7)%	(83.7 ± 1.7)%	−0.87	(0.82 ± 0.26)
DWI	142	15	6	[160, 160, 32]	(80.8 ± 6.6)%	(82.0 ± 5.9)%	−0.87	(0.70 ± 0.27)
CT	34	5	5	[96, 128, 96]	(80.7 ± 8.2)%	(77.9 ± 5.2)%	−0.97	(1.12 ± 0.40)

*Each dataset contains N_train_ training volumes, N_val_ validation volumes and N_test_ test samples. The voxel dimension of all volumes in the training and test dataset is fixed to the reported volume shape. Average and weighted Dice scores are reported according to Equations (4) and (5), respectively. The Pearson correlation coefficient between average Dice score and the uncertainty metric CV, obtained from dropout sampling, is listed for each imaging modality. The average symmetric surface distance (ASSD) in millimeters is reported as mean±s.d., where the mean and standard deviation (s.d.) are computed over all samples from the test dataset and averaged over all anatomical structures*.

Here, *P*_*s*_(*x*) is the softmax output of the network at voxel position *x* and *T*_*s*_(*x*) is the ground truth at the same position. We use the categorical cross-entropy loss to alleviate convergence problems when using solely the Dice loss (27). In principle, the influence of cross-entropy and Dice loss can be additionally weighted, but we found little influence on performance and therefore omit additional weighting. We train the CNNs for up to 400 epochs and abort the training process, if the validation loss does not improve for 100 epochs. The performance of the models is evaluated on separate test datasets.

### 2.3. MRI/CT Databases: Preprocessing and Label Generation

For training of the CNNs we use two large database of MRI and CT brain scans acquired on healthy patients. The acquisition parameters are listed in Supplementary Table 3. The first database contains 530 scans of healthy patients for which MPRAGE, FLAIR and DWI scans are available. The MPRAGE contrast was used to generate training labels using FreeSurfer 6.0 (4). The FreeSurfer labels were mapped to 27 segmentation classes using the mapping strategy described in (25). The resulting labels are in the following considered the ground truth and subsequently coregistered to the corresponding FLAIR and DWI scans.

After coregistration we manually checked for a proper alignment of the segmentation masks to the FLAIR or DWI volume. Out of the initial database with 530 cases, we select 124 (FLAIR) and 163 (DWI) volumes for training, validation and testing. We thus removed a large fraction of cases from the database. This is due to the limited fidelity of the coregistration process and because we observe that a smaller, yet higher quality database leads to better segmentation performance. For the MPRAGE contrast no further coregistration was necessary and we therefore manually selected a large fraction of 522 out of 530 volumes, with high-quality FreeSurfer segmentations, for training.

The second database contains 60 healthy patients for which both MPRAGE and CT brain scans are available. Again we coregister MPRAGE and CT volumes to each other and use FreeSurfer on the MPRAGE scans to obtain training labels for both imaging modalities. By manually checking the alignment of the segmentation mask to the CT volume we selected 41 volumes for training, validation, and testing. Here, we also manually corrected minor coregistration errors to keep most of the available samples for training.

In order to evaluate the achievable segmentation performance on the different imaging modalities, we perform two separate studies, which are presented in the results section. In the first study, we use all available samples on each modality for training, validation and testing and in the second study we remove scans from the larger databases to ensure equally-sized training datasets. The number of resulting training, validation and test samples for all modalities is listed in Tables 1, 2 for both studies. In order to ensure comparability among modalities, we decided to use the same patients/volumes for testing on the MRI modalities. This constrained the size of the test dataset to the intersection of the three MRI datasets, which includes in total six patients/volumes.

**Table 2:** Parameters of training and test datasets and segmentation scores on all four imaging modalities using approximately equally-sized training sets.

**Modality**	***N*_train_**	***N*_val_**	***N*_test_**	**Volume shape**	**Average** $D_{A}$	**Weighted** $D_{V}$	**ASSD [mm]**
MPRAGE	39	3	6	[128, 128, 128]	(83.6 ± 7.2)%	(86.6 ± 2.0)%	(0.81 ± 0.31)
FLAIR	39	3	6	[128, 128, 128]	(80.0 ± 8.8)%	(82.6 ± 2.1)%	(0.94 ± 0.38)
DWI	39	3	6	[160, 160, 32]	(78.9 ± 7.8)%	(80.7 ± 5.8)%	(0.71 ± 0.28)
CT^*a*^	34	5	5	[96, 128, 96]	(80.7 ± 8.2)%	(77.9 ± 5.2)%	(1.12 ± 0.40)

*Each dataset contains N_train_ training volumes, N_val_ validation volumes, and N_test_ test samples. The voxel dimension of all volumes in the training and test dataset is fixed to the reported volume shape. Average and weighted Dice scores are reported according to Equations (4) and (5), respectively. The average symmetric surface distance (ASSD) in millimeters is reported as mean ± s.d., where the mean and standard deviation (s.d.) are computed over all samples from the test dataset and averaged over all anatomical structures. ^a^Same model as reported in Table 1*.

### 2.4. Segmentation Performance Metrics

We use several metrics to quantify the segmentation performance of our models. As an overlap-based metric, we use the Dice score $D_{s}$, associated with the anatomical structure *s* ∈ S, as the performance metric:

(3) $$D_{s}=\frac{2\sum_{x}S_{s}(x)T_{s}(x)}{\sum_{x}S_{s}(x)+T_{s}(x)}.$$

Here, *S*_*s*_(*x*) is the hard segmentation mask, given in Equation (1), in one-hot encoding format. To compare the overall performance, we introduce two additional metrics: The average Dice score:

(4) $$D_{A}=\sum_{s\in S}D_{s},$$

and a volume-weighted Dice score:

(5) $$D_{V}=\frac{1}{V}\sum_{s\in S}V_{s}D_{s}.$$

Here, $V_{s}$ is the volume of the structure *s* and *V* is the total volume of all anatomical structures $V=\underset{s\in S}{\sum }V_{s}$. The background label is not included in the average and the volume-weighted Dice score and the volumes are computed from the segmentation masks.

In addition to the overlap-based Dice similarity metric, we also report the average symmetric surface distance (ASSD) in millimeters:

(6) $$\text{ASSD}(A,B)=\frac{1}{\left|A|+|B\right|}\left(\sum_{a\in A}D_{B}(a)+\sum_{b\in B}D_{A}(b)\right).$$

Here, *A* and *B* are the surfaces of ground-truth and predicted anatomical features, respectively. *D*_*B*_(*a*) is the minimal distance between surface *B* and a given surface voxel *a* ∈ *A* and *D*_*A*_(*b*) is the minimal distance between surface *A* and a given surface voxel *b* ∈ *B*. Further, |*A*| and |*B*| are the number of surface voxels.

We compute the ASSD, for each anatomical structure, using the hard segmentation mask *S*_*s*_(*x*) and the ground truth mask *T*_*s*_(*x*) with the python module Scipy (31). Apart from the individual ASSDs for each anatomical structure, we also report the average value of the ASSD to reduce the metric into a single quantity.

### 2.5. Uncertainty Quantification

A common challenge for automatic segmentation tools is uncertainty quantification or quality control of the segmentation output. Low quality segmentation can occur, for example, due to corrupted input volumes, acquisition artifacts, unrecognized pathologies, or in general due to input volumes outside the training distribution. The incorporation of a direct quality control method, into the segmentation process, is therefore highly desirable.

The softmax output of neural networks, however, does not directly provide credible information on the certainty associated with the assigned labels (32). Instead the authors of (32) proposed to use the dropout layers of the network during prediction to make the network output stochastic. By switching some nodes off at random, we can generate a set of *N* Monte Carlo (MC) samples $P_{s}^{1},\ldots ,P_{s}^{N}$ from the network output. The distribution of the MC samples can subsequently be used to gauge the certainty of the assigned labels. Recently, this approach has been successfully applied to brain segmentation on *T*_1_-weighted MRI scans in (29) and we follow their methodology to equip our segmentation pipeline with a credibility metric.

To integrate dropout sampling into our segmentation pipeline we keep the dropout layers of the networks active after training. We generate *N* = 15 MC segmentation samples using the, now stochastic, output of the network. The dropout rate is here fixed to *r* = 0.2 for all neural networks. The final segmentation map is obtained by adding the softmax outputs of all MC samples and then applying the argmax function:

(7) $$S(x)=\text{arg }\underset{s}{\text{max}}\sum_{i=1}^{N}P_{s}^{i}(x).$$

To gauge the quality of the segmentation, we use the coefficient of variation *CV*_*s*_ of anatomical volumes over the MC samples. This metric was introduced in (29) and reads:

(8) $$CV_{s}=\frac{\sigma_{s}}{\mu_{s}}.$$

Here, σ_*s*_ is the variance of the anatomical volumes between MC samples and μ_*s*_ is the mean volume. To reduce the uncertainty measure to a single quantity *CV* we additionally average the coefficient of variation over all segmented structures:

(9) $$CV=\sum_{s\in S}CV_{s}.$$

## 3. Results

### 3.1. Segmentation Performance

#### 3.1.1. Modality-Dependent Segmentation Performance Using All Training Samples

**Figure 2:**
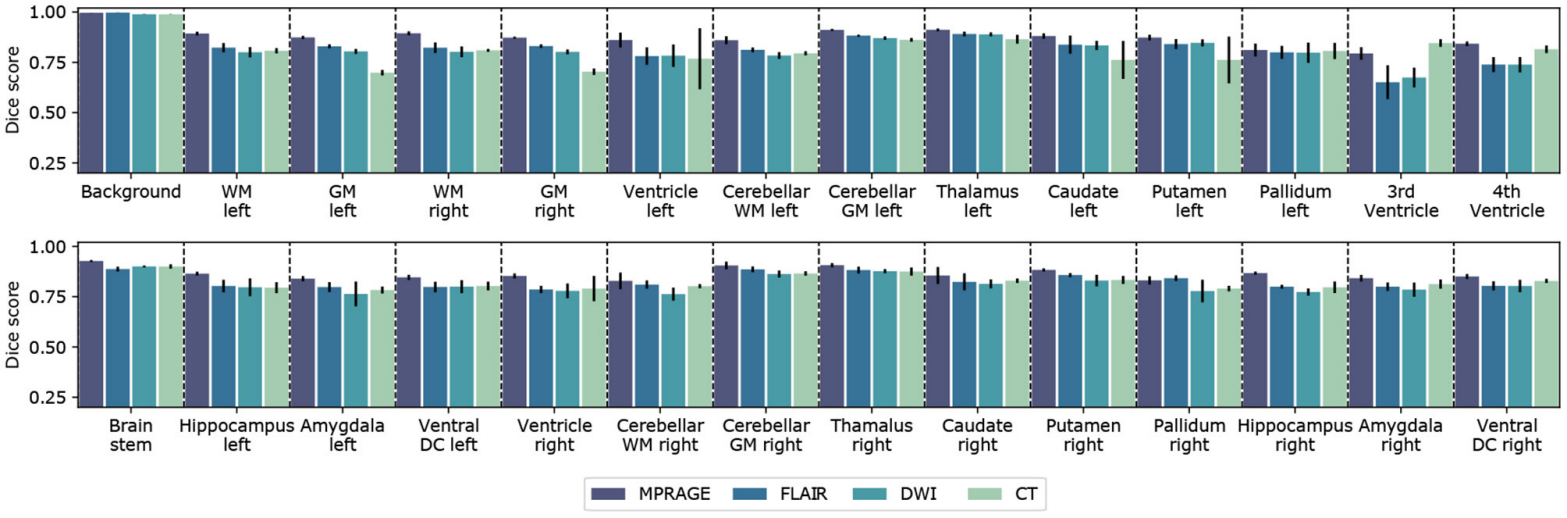
Segmentation performance for different imaging modalities. Barplot of the Dice score for all 27 segmented anatomical structures and including background. For each imaging modality a separate neural network was trained and evaluated. Error bars extend from the lower to upper quartile values of the data. The Dice scores were computed from the test datasets for the corresponding imaging modality, which included 6 (MPRAGE), 6 (DWI), 6 (FLAIR), and 5 (CT) samples, respectively. All parameters of the trained CNNs are summarized in Table 1.

In Figure 2, we compare the performance of the segmentation networks on the different imaging modalities for all 27 labeled structures and the background. The reported Dice scores represent the mean over all scans from the test set and the error bars extend from the lower to the upper quartile of values. We further collect all resulting metrics for the different imaging modalities in Table 1. This table also includes the ASSD, averaged over all anatomical structures.

We find that the best segmentation results are obtained for *T*_1_-weighted, MPRAGE scans for almost all investigated anatomical structures. This is also expressed by the best average Dice score $D_{A}$(MPRAGE) = (86.7 ± 4.1)% and the best volume-weighted Dice score $D_{V}$(MPRAGE) = (88.7 ± 0.8)%. Second-best performance is achieved on the FLAIR contrast. Here, the average Dice score is $D_{A}$(FLAIR) = (81.9 ± 6.7)% and the volume-weighted Dice score is $D_{V}$(FLAIR) = (83.7 ± 1.7)%. However, the difference to the performance on the DWI contrast with $D_{A}$(DWI) = (80.8 ± 6.6)% and $D_{V}$(DWI) = (82.0 ± 5.9)% is small.

For CT scans, we find that the segmentation performance is strongly structure-dependent: The low signal contrast between gray and white matter limits to some extent the accuracy of the segmentation, especially of the gray matter regions. At the same time, the segmentation of structures like e.g. ventricles, the putamen or the hippocampus can be performed with high accuracy. We find an average Dice score $D_{A}$(CT) = (80.7 ± 8.2)% on the CT dataset and the volume-weighted Dice score is $D_{V}$(CT) = (77.9 ± 5.2)%. The significant reduction in the volume-weighted score is due to the large volume fraction of gray and white matter.

In terms of the surface distances, we find the lowest ASSD, averaged over all anatomical structures, for the MPRAGE scans with 0.67 ± 0.23 mm. The ASSD for the FLAIR scans is slightly higher with 0.82 ± 0.26mm and exceeds the value for the DWI scans with 0.70 ± 0.27 mm. Again, the lowest performance is found for the CT scans with an ASSD of 1.12 ± 0.40 mm. In general, both similarity and distance-based metrics lead to a consistent quality assessment of the segmentation performance.

#### 3.1.2. Modality-Dependent Segmentation Performance Using Equally-Sized Training Sets

In the previous section, we analyzed the achievable segmentation performance for the different imaging modalities by using all available scans in our database. As a consequence, the number of training samples *N*_train_ is significantly imbalanced same for the different modalities (see Table 1). In order to compare segmentation performance under equally-sized training data sets, we retrained all MRI-related models with a common number of samples (*N*_train_ = 39, *N*_val_ = 3, *N*_test_ = 6). Consequently, for the MPRAGE, FLAIR and DWI scans the number of training samples is reduced by 426, 68, 103 samples, respectively. We use scans from the same subjects out of the first database to make the comparison as fair as possible. We compare the segmentation performance to the existing CT model, which has a similar number of training samples s(*N*_train_ = 34).

In Table 2, we list the resulting average and volume-weighted Dice scores of the new models on the test sets. We observe, that due to the reduction of training samples the model performance is consistently reduced by approximately 1 − 2%. Nevertheless, the ranking of model performance on different modalities remains as in the previous section.

#### 3.1.3. Dependence of Segmentation Performance on the Number of Training Samples

**Figure 3:**
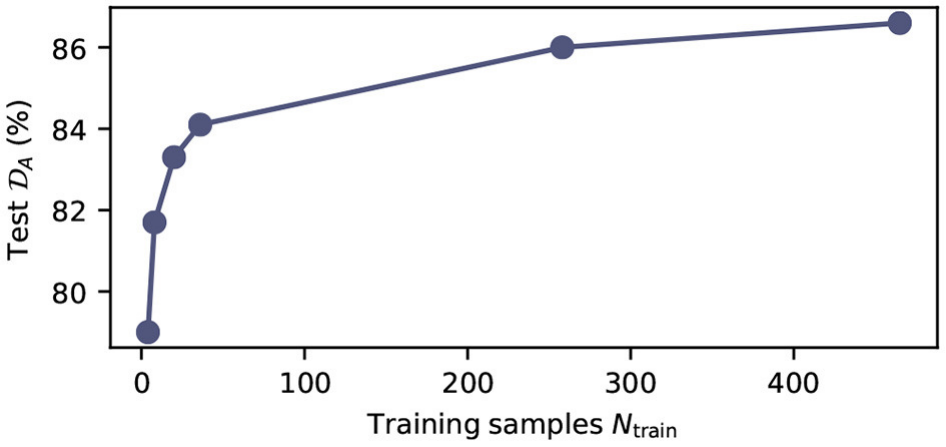
Model performance as function of the number of training samples. Average test Dice score $D_{A}$ on the MPRAGE dataset (*N*_test_ = 6) as function of training samples *N*_train_. All models were trained with the same hyperparameters as summarized in Supplementary Table 3. The exact number of training samples for the plotted data points is *N*_train_ ∈ {4, 8, 20, 258, 465}.

To further quantify the relation between model performance and the number of available training samples, we trained in total six networks, with varying numbers of training samples *N*_train_ ∈ {4, 8, 20, 258, 465}, on the MPRAGE contrast. The training samples were chosen randomly from the training database. In Figure 3, we show the average Dice score on the test set as a function of the number of training samples (*N*_test_ = 6 for all models). We find that the test score improves monotonically with the number of available training samples and increases from (79.0 ± 8.7%) for the smallest training dataset (*N*_train_ = 4) to (86.7 ± 4.1%) for the largest training dataset (*N*_train_ = 489). When training with *N*_train_ > 100, the gain in performance levels off significantly, but does not reach saturation. All models were trained with identical hyperparameters, as described in the Methods section.

### 3.2. Example Segmentations

**Figure 4:**
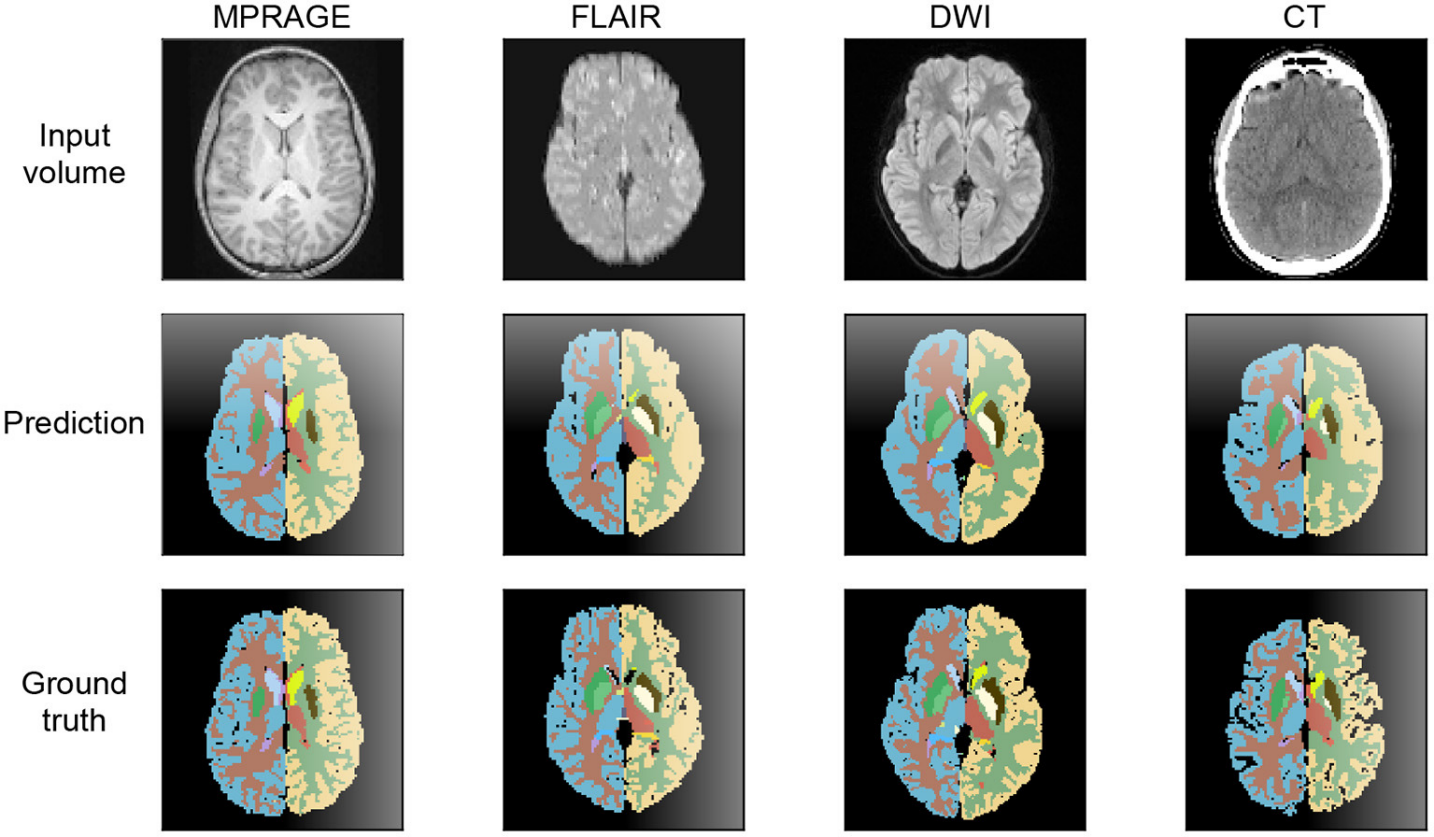
Axial view at the level of the basal ganglia of brain segmentations on MPRAGE, FLAIR, DWI, and CT. The MRI were obtained from the same patient, the CT image stems from a different patient. The thalamus, the nucleus lentiformis, the nucleus caudatus, and the cortical ribbon are well-demarcated on all contrast. The segmentation of the cortical ribbon on CT and DWI, where the white matter–gray matter (WM-GM) contrast is low, is less detailed compared to MPRAGE, but still of good quality.

In Figure 4, we show exemplary input slices, ground-truth labels and the prediction of our segmentation networks for each of the four imaging modalities in the axial view. For the MPRAGE, DWI and FLAIR modalities the segmentation was performed on the same patient and approximately the same slice location is displayed. Exact overlapping of slices is not possible, because the scans are not coregistered to each other. For the CT scan a separate patient was selected from the test dataset of the second database. All predictions are taken from models, which were trained with all available training samples.

The example segmentation clearly show that the gray and white matter boundaries are captured best on the *T*_1_-weighted MPRAGE contrast. Here, even fine structures are properly distinguished. On the DWI and FLAIR contrast gray and white matter are segmented with lower level of detail and with lower fidelity. Due to the significantly reduced signal contrast, the gray and white matter segmentation on the CT scans displays a further reduction in performance. In terms of anatomical structures other than gray and white matter, the CT segmentation provides excellent results. This is especially the case for the ventricles, which are segmented more precisely than on the FLAIR and DWI scans.

### 3.3. Uncertainty Quantification

**Figure 5:**
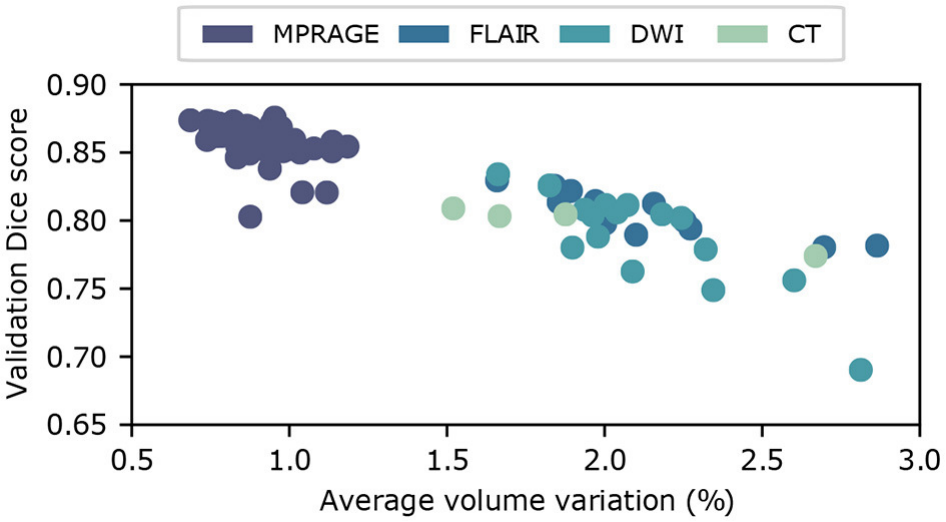
Uncertainty estimation using dropout sampling. Scatter plot of the average Dice score $D_{A}$ vs. the coefficient of variation *CV*. Each scatter point corresponds to a input volume from the test dataset. Coefficients of variation are obtained from *N* = 15 MC samples. The strong correlation between $D_{A}$ and *CV* demonstrates that *CV* is a good measure of segmentation quality. Pearson correlation coefficients, derived separately for each imaging modality, are summarized in Table 1.

In Figure 5, we show the relationship between uncertainty metric *CV* and the average Dice score $D_{A}$, derived from the ground truth labels, for volumes from the test set. Here, we combine the results for all imaging modalities. We clearly observe a strong correlation between *CV*_*s*_ and Dice scores, which indicates that *CV* is in fact a good metric to gauge the quality of the segmentation. The Pearson correlation coefficients are *C*_MPRAGE_ = −0.91, *C*_FLAIR_ = −0.87, *C*_DWI_ = −0.85, and *C*_CT_ = −0.98, for the corresponding imaging modalities. As a consequence, we integrate the dropout sampling as an optional processing step into our pipeline, which warns the user if the coefficient of variation *CV* for the requested segmentation exceeds 1.0% for MPRAGE and 2.5% for FLAIR, DWI and CT contrasts, respectively.

## 4. Discussion

In this work, we investigated the segmentation quality that can be achieved using convolutional neural networks on various MR/CT imaging modalities. In a first study with in total 853 MR/CT scans, we find that *T*_1_-weighted images provide the best segmentation results. This finding agrees with our naive expectation, because the *T*_1_-weighted MPRAGE scans provide the best gray-to-white matter contrast and the ground truth labels were generated on this contrast. Further, the largest dataset for training was available for this contrast. Nevertheless, also FLAIR, DWI and even CT scans can be segmented with excellent results when using current state of the art deep neural networks. In case of CT scans, we observe that segmentation quality is dependent on the anatomical structure. While gray and white matter segmentation is challenging, due to low signal contrast, the performance on ventricles, putamen, pallidum, and brain stem reaches or exceeds the performance achieved on the MRI contrasts.

Because our database of available training samples in the first study is imbalanced across the different modalities, we performed a second study with approximately equally-sized training sets to facilitate a fairer comparison. We found that the general ranking of the achievable segmentation performance remains the same, but that the difference between the performances reduces. Additionally, we investigated the scaling behavior of model performance with the number of available training samples on the MPRAGE contrast.

Finally, we implemented an uncertainty estimator, using dropout sampling as introduced in (29), to gauge the quality of the generated segmentation labels. We observe a strong correlation between our uncertainty metric, the coefficient of volume variation *CV*, and the quality of the segmentation derived from the ground truth labels. This is the case for all imaging modalities. Consequently, we incorporate the uncertainty metric *CV* in our segmentation pipeline to identify faulty input volumes or low-quality segmentation.

Based upon the presented results, several improvements and further investigations that extend the applicability of our segmentation pipeline can be envisioned: In our work, we use input volumes with ~2 million voxels, which is limited by the available memory on our GPU. In the future, it would be desirable to scale this number up by one order of magnitude in order to directly process entire 3D brain scans with an isotropic resolution of 1mm, which results in input volumes with approximately 20 million voxels. This could be achieved by a combination of more memory-efficient network architectures, ensembles of smaller segmentation networks, which only address a subset of labels, or via improvements in GPU hardware.

In addition, our investigation on the achievable segmentation performance on different imaging modalities is only the first step toward reliable segmentation tools for DWI, FLAIR, and CT volumes. As a next step, the segmentation performance should also be quantified relative to manual label maps from human experts. This could be done by performing the manual annotation on MPRAGE scans and transferring the labels over to the other modalities, as it was done in our study with FreeSurfer labels. Alternatively, the annotations could also be directly added to the DWI, FLAIR, and CT volumes. This was recently done for CT scans (33) and would alleviate the systematic errors introduced by non-perfect coregistration.

Based on performance, flexibility, and processing speed, CNNs already now represent a valuable tool for automated anatomical segmentation. In our view, however, the most significant obstacle to the broad applicability of segmentation CNNs is the limited generalizability to different acquisition parameters and MRI/CT scanners. To train networks that generalize very well, the generation and distribution of large structured databases of MRI and CT scans, acquired on various scanners and imaging contrasts, is highly desirable. In addition, further research on the combination or improvement of methods, such as lifelong learning (34) or advanced data augmentation (35) is necessary. In terms of data augmentation, generative models, such as generative adversarial networks (GANs) or variational autoencoders (VAEs) could be used to generate large databases of synthetic MRI/CT scans. These databases could subsequently be used to enhance training.

## Data Availability Statement

The data analyzed in this study is subject to the following licenses/restrictions: datasets are not public due to data protection. Request to access these datasets should be directed to federau@biomed.ee.ethz.ch.

## Ethics Statement

The studies involving human participants were reviewed and approved by Ethikkommission Nordwest- und Zentralschweiz. Written informed consent for participation was not required for this study in accordance with the national legislation and the institutional requirements.

## Author Contributions

JZ and SP implemented and trained the neural networks. JZ wrote the manuscript with input from all other authors. All authors were involved in the preparation of the dataset.

## Conflict of Interest

The authors declare that the research was conducted in the absence of any commercial or financial relationships that could be construed as a potential conflict of interest.
