# Supplementary material for: Multi-Modal Segmentation of 3D Brain Scans Using Neural Networks
Source: Front Neurol. 2021 Jul 14;12:653375. doi: 10.3389/fneur.2021.653375 (PMC8318570; doi:10.3389/fneur.2021.653375)
Supplement: Supplementary file 1 [file Table_1.pdf]

ORIGINAL ARTICLE

Supplementary Information for: Multi-modal segmentation of 3D brain scans using neural networks

Jonathan Zopes, Moritz Platscher, Silvio Paganucci, Christian Federau

The Supplementary Information contain four tables with additional information on the segmented anatomical structures, on the detailed parameters of the neural networks and on the acquisition parameters of the MR scans.

Segmentation index	Anatomical structure
1	Cortical White Matter Left
2	Cortical Grey Matter Left
3	Cortical White Matter Right
4	Cortical Grey Matter Right
5	Lateral Ventricle Left
6	Cerebellar White Matter Left
7	Cerebellar Grey Matter Left
8	Thalamus Left
9	Caudate Left
10	Putamen Left
11	Pallidum Left
12	Third Ventricle
13	Fourth Ventricle
14	Brainstem
15	Hippocampus Left
16	Amygdala Left
17	Ventral DC Left
18	Lateral Ventricle Right
19	Cerebellar White Matter Right
20	Cerebellar Grey Matter Right
21	Thalamus Right
22	Caudate Right
23	Putamen Right
24	Pallidum Right
25	Hippocampus Right
26	Amygdala Right
27	Ventral DC Right

TABLE S1 **List of all segmented anatomical structures.** Background labels are assigned with the index 0.

Anatomical structure	MPRAGE	FLAIR	DWI	CT
Cortical WM Left	(0.52 ± 0.02) mm	(0.83 ± 0.11) mm	(0.79 ± 0.12) mm	(1.24 ± 0.04) mm
Cortical GM Left	(0.61 ± 0.04) mm	(0.79 ± 0.05) mm	(0.70 ± 0.03) mm	(0.99 ± 0.04) mm
Cortical WM Right	(0.51 ± 0.03) mm	(0.80 ± 0.09) mm	(0.79 ± 0.14) mm	(1.28 ± 0.03) mm
Cortical GM Right	(0.62 ± 0.04) mm	(0.79 ± 0.05) mm	(0.71 ± 0.05) mm	(1.05 ± 0.04) mm
Lateral Ventricle Left	(0.53 ± 0.11) mm	(0.75 ± 0.23) mm	(0.58 ± 0.21) mm	(1.12 ± 0.65) mm
Cerebellar WM Left	(0.84 ± 0.20) mm	(1.11 ± 0.14) mm	(1.08 ± 0.17) mm	(1.49 ± 0.13) mm
Cerebellar GM Left	(0.82 ± 0.08) mm	(1.03 ± 0.07) mm	(0.83 ± 0.08) mm	(1.62 ± 0.19) mm
Thalamus Left	(0.71 ± 0.03) mm	(0.83 ± 0.11) mm	(0.56 ± 0.10) mm	(1.21 ± 0.34) mm
Caudate Left	(0.60 ± 0.09) mm	(0.79 ± 0.45) mm	(0.51 ± 0.12) mm	(1.43 ± 1.09) mm
Putamen Left	(0.66 ± 0.08) mm	(0.74 ± 0.13) mm	(0.58 ± 0.09) mm	(1.35 ± 0.73) mm
Pallidum Left	(0.91 ± 0.19) mm	(0.94 ± 0.22) mm	(0.63 ± 0.25) mm	(0.95 ± 0.21) mm
Third Ventricle	(0.50 ± 0.18) mm	(0.99 ± 0.61) mm	(0.82 ± 0.48) mm	(0.61 ± 0.23) mm
Fourth Ventricle	(0.48 ± 0.04) mm	(0.79 ± 0.13) mm	(0.84 ± 0.23) mm	(0.81 ± 0.17) mm
Brainstem	(0.62 ± 0.08) mm	(0.90 ± 0.16) mm	(0.70 ± 0.12) mm	(0.97 ± 0.17) mm
Hippocampus Left	(0.63 ± 0.08) mm	(0.79 ± 0.17) mm	(0.56 ± 0.25) mm	(1.07 ± 0.19) mm
Amygdala Left	(0.69 ± 0.10) mm	(0.82 ± 0.14) mm	(0.65 ± 0.18) mm	(0.96 ± 0.19) mm
Ventral DC Left	(0.68 ± 0.10) mm	(0.83 ± 0.16) mm	(0.62 ± 0.17) mm	(0.96 ± 0.14) mm
Lateral Ventricle Right	(0.54 ± 0.14) mm	(0.76 ± 0.27) mm	(0.61 ± 0.15) mm	(0.97 ± 0.18) mm
Cerebellar WM Right	(1.11 ± 0.52) mm	(1.14 ± 0.30) mm	(1.25 ± 0.53) mm	(1.44 ± 0.14) mm
Cerebellar GM Right	(0.86 ± 0.18) mm	(0.98 ± 0.11) mm	(0.88 ± 0.18) mm	(1.52 ± 0.16) mm
Thalamus Right	(0.80 ± 0.24) mm	(0.81 ± 0.13) mm	(0.65 ± 0.11) mm	(1.08 ± 0.26) mm
Caudate Right	(0.74 ± 0.33) mm	(0.69 ± 0.12) mm	(0.66 ± 0.14) mm	(1.02 ± 0.25) mm
Putamen Right	(0.66 ± 0.06) mm	(0.73 ± 0.10) mm	(0.68 ± 0.19) mm	(1.08 ± 0.21) mm
Pallidum Right	(0.74 ± 0.15) mm	(0.78 ± 0.11) mm	(0.67 ± 0.23) mm	(1.05 ± 0.05) mm
Hippocampus Right	(0.62 ± 0.04) mm	(0.84 ± 0.08) mm	(0.60 ± 0.09) mm	(1.04 ± 0.17) mm
Amygdala Right	(0.82 ± 0.20) mm	(0.80 ± 0.08) mm	(0.68 ± 0.22) mm	(0.99 ± 0.17) mm
**Average over all structures**	(0.68 ± 0.09) mm	(0.80 ± 0.12) mm	(0.63 ± 0.25) mm	(1.12 ± 0.40) mm

TABLE S2 Average symmetric surface distance (ASSD) for all investigated anatomical structures. We report mean ± standard deviation of the ASSD. Mean and standard deviation are computed for the samples from the test dataset. The last row reports the average over all anatomical structures, as also reported in Tab. 1 in the main text.

	MPRAGE	FLAIR	DWI	CT
Input dimension	128<br>128<br>128	128<br>128<br>128	160<br>160<br>32	96<br>128<br>96
Initial feature maps F	32	32	32	32
Depth of network D	4	4	4	4
Bottleneck layers B	2	2	2	2
Conv. kernel size K	(3, 3, 3)	(3, 3, 3)	(3, 3, 3)	(3, 3, 3)
Number of parameters	22.58M	22.58M	22.57M	22.58M

TABLE S3 Summary of CNN parameters. The architecture of the CNNs follows the U-Net architecture: The initial number of feature maps F, after the first convolutional layer, is doubled in each encoder block and halved in each of the decoder blocks. The depth D of the network describes the number of down- or upsampling operations. For example, the network shown in Figure 1 in the main text represents a network of depth $D = 2$. The number of bottleneck layers B, specifies how many convolutional layers are part of the bottleneck. In the bottleneck each convolutional layer is followed by batch normalization and rectified linear activations.

Parameter	MPRAGE	FLAIR	DWI
Repetition time T_R (ms)	1938 ± 480	7999 ± 711	6926 ± 781
Echo time T_E (ms)	3.25 ± 0.54	114 ± 28	73 ± 16
Inversion time T_I (ms)	945 ± 120	2362 ± 120	N.A.
Acquisition matrix (range)	[224 – 288, 184 – 288]	[256 – 320, 168 – 320]	[96 – 200, 96 – 200]
Slice thickness (mm)	1.01 ± 0.06	3.88 ± 0.53	3.69 ± 0.64
Pixel bandwidth (Hz)	201 ± 19	292 ± 45	1018 ± 211
Field strength 3T / 1.5T (% of cases)	95 %/5 %	100 %/0 %	92 %/8 %

TABLE S4 Summary of MR acquisition parameters. The repetition time (T_R), echo time (T_E), slice thickness and pixel bandwidth are reported as $\mu \pm \sigma$. Here, μ is the average value of the distribution of the parameter and σ is the corresponding standard deviation.

#	count	$B_0[T]$	$T_R(s)$	$T_E(ms)$	$T_I(s)$	Ac. Matrix	Slc. Thickness	Bandwidth (Hz)
0 (GE)	10	3.0	0.01 ± 0.0	3.71 ± 0.02	0.45 ± 0.0	[256, 256]	1.2 ± 0.0 mm	195.31 ± 0.0
1 (GE)	15	3.0	0.01 ± 0.0	3.53 ± 0.02	0.45 ± 0.0	[256, 256]	1.19 ± 0.05 mm	217.03 ± 0.0
2 (S)	6	1.5	2.7 ± 0.0	5.03 ± 0.0	0.95 ± 0.0	[256, 256]	1.0 ± 0.0 mm	130.0 ± 0.0
3 (S)	4	1.5	2.7 ± 0.0	4.93 ± 0.0	0.95 ± 0.0	[256, 256]	1.0 ± 0.0 mm	140.0 ± 0.0
4 (S)	29	3.0	2.13 ± 0.15	2.93 ± 0.59	0.96 ± 0.05	[240, 256]	1.0 ± 0.0 mm	216.0 ± 19.0
5 (S)	353	3.0	1.94 ± 0.13	3.26 ± 0.3	0.99 ± 0.03	[184, 288]	1.01 ± 0.05 mm	199.0 ± 4.3
6 (S)	38	3.0	2.3 ± 0.0	2.45 ± 0.3	0.9 ± 0.0	[240, 256]	1.01 ± 0.05 mm	212.0 ± 18.0
7 (S)	4	3.0	1.68 ± 0.21	2.7 ± 0.45	0.92 ± 0.05	[256, 256]	1.0 ± 0.0 mm	200.0 ± 0.0
8 (S)	12	1.5	2.32 ± 0.23	4.65 ± 0.23	0.95 ± 0.0	[256, 256]	1.0 ± 0.0 mm	145.0 ± 9.1
9 (S)	7	1.5	2.2 ± 0.0	4.52 ± 0.0	0.95 ± 0.0	[256, 256]	1.0 ± 0.0 mm	150.0 ± 0.0
10 (S)	44	3.0	2.29 ± 0.06	2.92 ± 0.25	0.9 ± 0.02	[240, 256]	0.99 ± 0.03 mm	229.0 ± 13.0

TABLE S5 Summary of scanner-specific MPRAGE acquisition parameters. $\mu \pm \sigma$ indicates mean and standard deviation, while brackets indicate a range. Manufacturers General electric (GE) and Siemens (S) are indicated.

#	count	$B_0[T]$	$T_R(s)$	$T_E(ms)$	Ac. Matrix	Slc. Thickness	Bandwidth (Hz)
0 (S)	2	1.5	6.0 ± 0.0	100.0 ± 0.0	[128, 128]	3.0 ± 0.0 mm	1184.0 ± 0.0
1 (S)	1	1.5	6.0	100.0	[116, 116]	3.0 mm	1221.0
2 (S)	3	3.0	8.07 ± 0.46	102.67 ± 2.31	[96, 128]	2.67 ± 0.58 mm	1472.0 ± 140.0
3 (S)	127	3.0	6.76 ± 0.52	64.51 ± 4.43	[144, 200]	3.98 ± 0.12 mm	921.0 ± 10.0
4 (S)	6	3.0	8.38 ± 0.7	96.83 ± 4.12	[96, 162]	2.5 ± 0.55 mm	1324.0 ± 340.0
5 (S)	1	3.0	5.8	74.0	[162, 162]	3.0 mm	1715.0
6 (S)	7	1.5	6.0 ± 0.0	100.0 ± 0.0	[128, 128]	3.0 ± 0.0 mm	1185.0 ± 0.0
7 (S)	3	1.5	6.0 ± 0.0	100.0 ± 0.0	[128, 128]	3.0 ± 0.0 mm	1185.0 ± 0.0
8 (S)	14	3.0	8.62 ± 0.27	103.21 ± 2.94	[96, 162]	2.07 ± 0.27 mm	1552.0 ± 110.0

TABLE S6 Summary of scanner-specific DWI acquisition parameters. $\mu \pm \sigma$ indicates mean and standard deviation, while brackets indicate a range.

#	count	$B_0[T]$	$T_R(s)$	$T_E(ms)$	$T_I(s)$	Ac. Matrix	Slc. Thickness	Bandwidth (Hz)
0 (S)	124	3.0	8.0 ± 0.71	113.81 ± 28.56	2.36 ± 0.12	[168, 320]	3.88 ± 0.53 mm	292.0 ± 45.0

TABLE S7 Summary of scanner-specific FLAIR acquisition parameters. Here, only a single scanner was used for acquisition.
